# Supplementary figures and images for: Macrophage and dendritic cell subset composition can distinguish endotypes in adjuvant-induced asthma mouse models
Source: PLoS One. 2021 Jun 1;16(6):e0250533. doi: 10.1371/journal.pone.0250533 (PMC8168852; doi:10.1371/journal.pone.0250533)

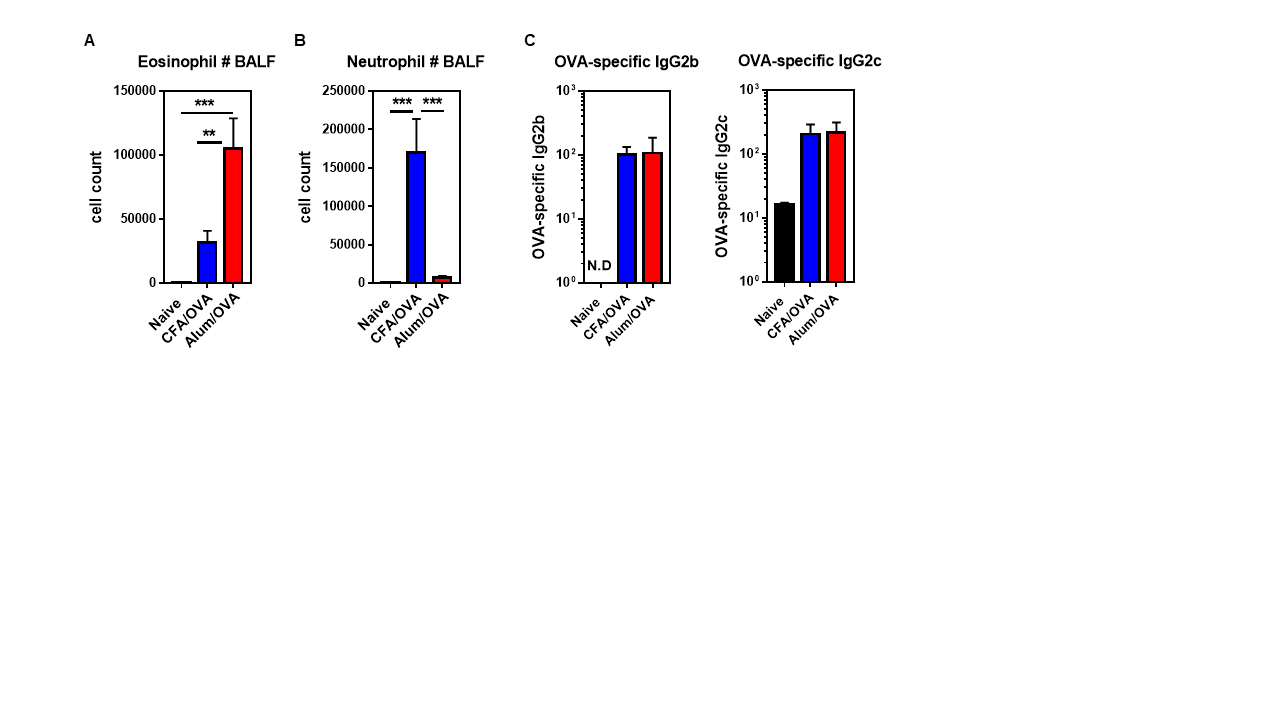

Supplement: S1 Fig — C57BL/6 mice were immunized as outlined in Fig 1A to induce neutrophilic (CFA/OVA) or eosinophilic (Alum/OVA) asthma. (A, B) Cells from bronchoalveolar lavage fluid (BALF) were stained for (A) eosinophils (live CD45+ CD19- CD11c- CD11b- Ly6G- Siglec-F+) and (B) neutrophils (live CD45+ CD19- CD11b+/lo Ly6G+). (C) Serum levels of OVA-specific IgG2b and IgG2c antibodies in the indicated groups were determined by ELISA. Combined data from four independent experiments are shown (naïve group n = 6 (C) or 12 (A, B) mice/group, experimental asthma groups n = 16 (C) or 17–18 (A, B) mice/group in total). (TIF) [file pone.0250533.s001.tif]

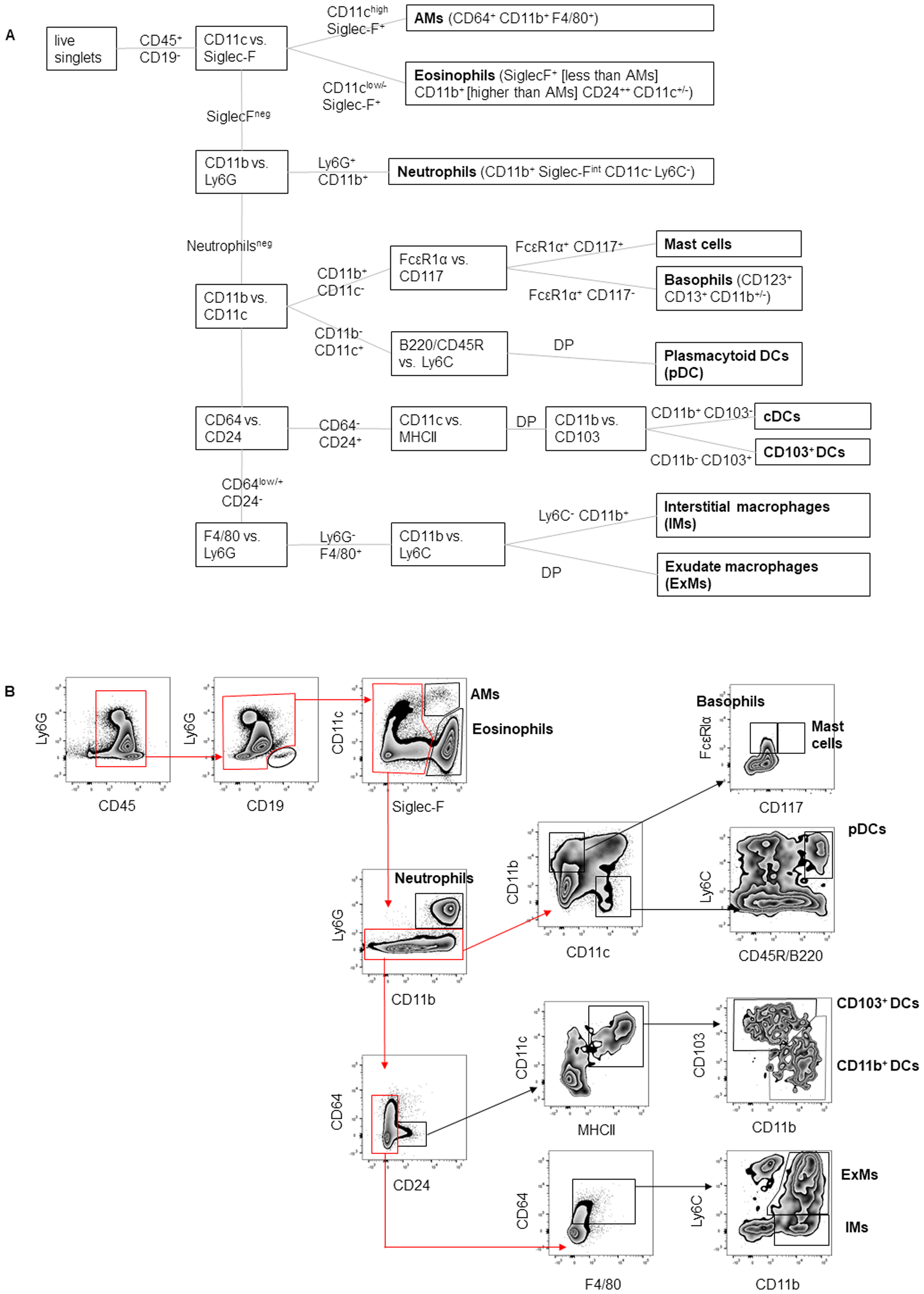

Supplement: S2 Fig — A graphic outline (A) and exemplary graphs (B) are given to illustrate the gating strategy employed to identify myeloid cells in the lung and BALF. Alveolar macrophages (AMs): live CD45+ CD19- Siglec-F+ Ly6G- CD11c+ cells; Basophils: live CD45+ CD19- Siglec-F- Ly6G- CD11b+ FcεRIα+ CD117- cells; Conventional DCs (cDC): live CD45+ CD19- Siglec-F- Ly6G- CD24+ CD11c+ MHC class II+ CD103- CD11b+ cells; CD103+ DCs: live CD45+ CD19- Siglec-F- Ly6G- CD24+ CD11c+ MHC class II+ CD103+ cells; Dendritic cells (all): live CD19- CD45+ Siglec-F- Ly6G- F4/80- CD64- CD24+ CD11c+ MHC class II+/-; Eosinophils: live CD45+ CD19- CD11c- CD11b- Ly6G- Siglec-F+ cells; Exudate macrophages (ExMs): live CD45+ CD19- Siglec-F- Ly6G- CD24- F4/80+ CD64+/- Ly6C+ CD11b+ cells; Interstitial macrophages (IMs): live CD45+ CD19- Siglec-F- Ly6G- CD24- F4/80+ CD64+/- Ly6C+ CD11b- cells; Neutrophils: live CD45+ CD19- CD11b+/lo Ly6G+ cells; Macrophages (all): live CD19- CD45+ Siglec-F- Ly6G- F4/80+ CD64+ cells; Mast cells: live CD45+ CD19- Siglec-F- Ly6G- CD11b+ FcεRIα+ CD117+ cells; Plasmacytoid dendritic cells: pDC, live CD19- CD45+ Siglec-F- Ly6G- CD11b- CD45R+ Ly6C+ cells. (TIF) [file pone.0250533.s002.tif]

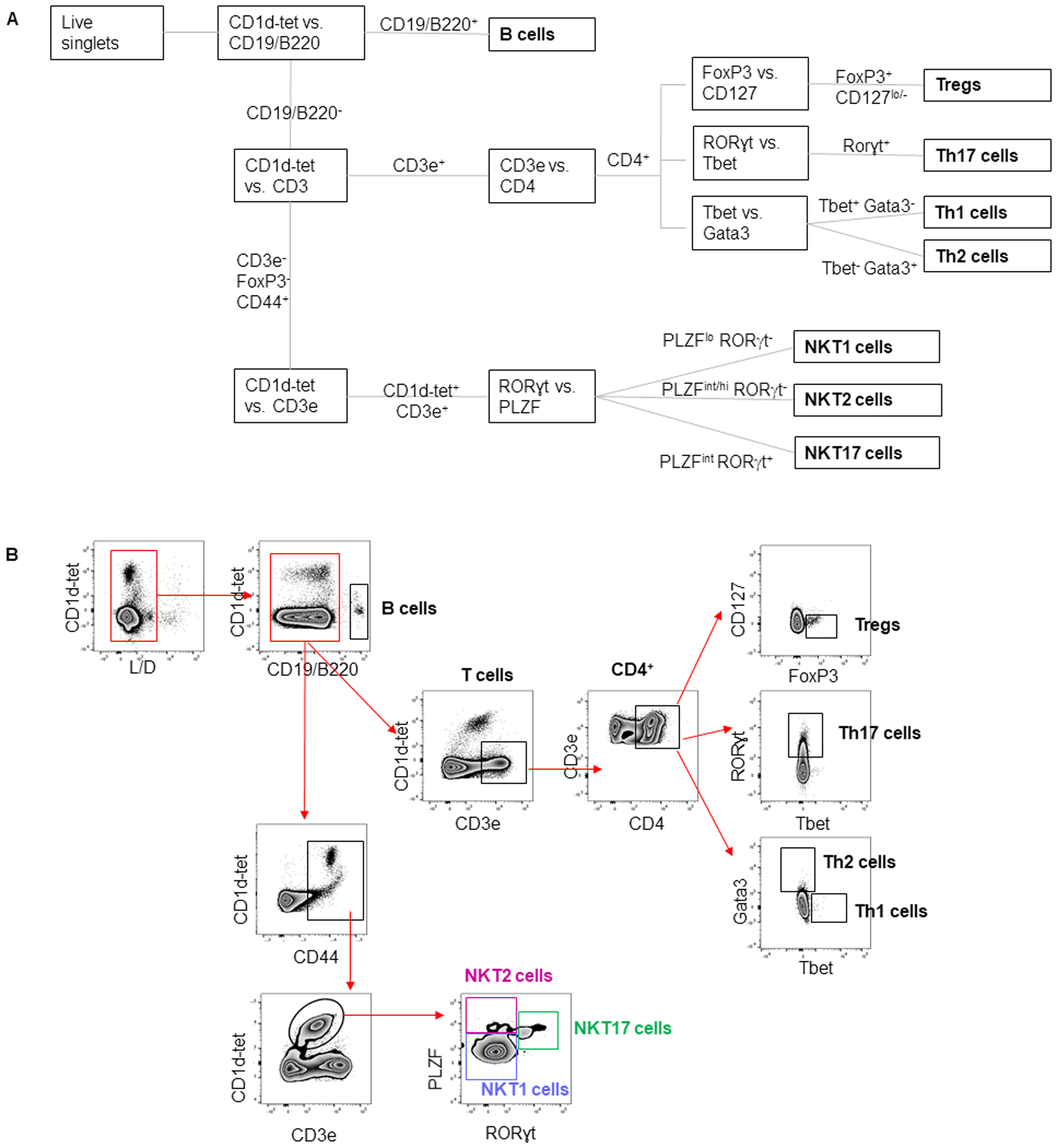

Supplement: S3 Fig — A graphic outline (A) and exemplary graphs (B) are given to illustrate the gating strategy employed to identify lymphoid cells in the lung and BALF. Th1 cells (live CD19/CD45R- CD3ε+ CD4+ Tbet+ cells), Th2 cells (live CD19/B220- CD3ε+ CD4+ Gata3+ cells), Th17 cells (live CD19/CD45R- CD3ε+ CD4+ RORɣt+ cells), and Tregs (live CD19/CD45R- CD3ε+ CD4+ CD127lo/- FoxP3+), iNKT cells (live CD19/CD45R- CD3ε+ CD1d/PBS57-tetramer+ cells) and its subsets in the lung NKT1 (PLZFlo RORγt-), NKT2 cells (PLZFint/hi RORγt-), NKT17 cells (PLZFint RORγt+) cells are shown. (TIF) [file pone.0250533.s003.tif]
