# Supplementary material for: Macrophage and dendritic cell subset composition can distinguish endotypes in adjuvant-induced asthma mouse models
Source: PLoS One. 2021 Jun 1;16(6):e0250533. doi: 10.1371/journal.pone.0250533 (PMC8168852; doi:10.1371/journal.pone.0250533)
Supplement: Attachment — Submitted filename: Ozkan_Point-to-point reply to reviewers.pdf [file pone.0250533.s004.pdf]

Asst. Prof. Gerhard Wingender Ph.D.
Izmir Biomedicine and Genome Center (IBG)
Dokuz Eylul University Health Campus
35340 Balcova/Izmir
Turkey
Email: gerhard.wingender@ibg.edu.tr

4 May 2021

RESPONSE TO THE REWIEVERS & EDITORS:

We thank the reviewer and editors for their time and efforts in their review, which helped us to improve the manuscript. Enclosed please find the revised version of the manuscript that takes into account the raised comments and suggestions, and that highlights the important changes in the text in yellow. Please find below our point-to-point reply to the issues raised.

Editors

1. Please ensure that your manuscript meets PLOS ONE's style requirements, including those for file naming.

Response: We followed PLOS ONE’s guidelines to our best knowledge and made the appropriated changes.

2. Please review your reference list to ensure that it is complete and correct. If you have cited papers that have been retracted, please include the rationale for doing so in the manuscript text, or remove these references and replace them with relevant current references. Any changes to the reference list should be mentioned in the rebuttal letter that accompanies your revised manuscript. If you need to cite a retracted article, indicate the article’s retracted status in the References list and also include a citation and full reference for the retraction notice.

Response: The reference list is complete and correct to the best of our knowledge. We checked the references on PubMed and none of them where flagged as retracted.

Reviewer #1

Comments:

1. Figure1C, naïve animal its self-shows 10% to 20% neutrophils in BALF and Lung, respectively. Can author explain why?

Response: The values we report (Fig.1C) for the relative frequency of neutrophils in the control BALF (9.4% +/- 2.3%) and lung (17% +/- 3.2%) appear to us in the range of what has been published before. For control lungs, Yipp *et al.* 2017 (PMID: 28626833, Fig.2B) or Barletta *et al.* 2012 (PMID: 21996427, abstract) reported that approx. 12% or 17% of the $CD45^+$ cells were neutrophils. For the BALF, we are not aware of publications that reported the relative frequency of neutrophils, as the data are commonly shown as absolute cell counts. To make our data more comparable, we now included the values of the absolute cell counts of eosinophils and neutrophils

in the BALF to the new supplemental figures S1A and S1B. These values are in line with previous findings (e.g. Bogaert *et al.* 2011, PMID: 21335522, Fig.1; Dejager *et al.* 2015, #25760421, Fig.1; Hosoki *et al.* 2016, #26086549, Fig.1). Neutrophils are highly mobile cells that are recruited to the naïve tissue, including the lung, also under resting conditions (e.g. Casanova-Acebes *et al.* 2018, #30282719, Fig.1D). Furthermore, it was shown that the relative frequency of neutrophils in particular tissues is influenced by genetics (strain background and gender, e.g. Hensel *et al.* 2019, #30353130, Fig.5). Additionally, the housing conditions strongly influence the commensal microbiota in mice (e.g. Ericsson *et al.* 2018, 29511208), which impacts their disease susceptibility not only in the intestine, but also in the lung (e.g. Barcik *et al.* 2020, #26086549; Gollwitzer *et al.* 2014, #24813249). However, such homeostatic aspects were not the focus of our study and would in our opinion not influence our conclusions from the adjuvant-induced asthma models. Altogether, we would therefore conclude that the values we report here appear not to be unusual and in line with previous work.

2a. Allergen specific mediastinal lymph node re-stimulation and cytokine (Th1/Th2/Th17) estimation in supernatant provides more appropriate idea to conclude the immune response generated during inflammation.

Response: Given that we studied allergen-induced airway inflammations, we feel that the analysis of the local immune response in the inflamed lung is more relevant to understand the immune response. We agree, however, that the analysis of the T cell cytokine response from the draining lymph nodes is more common and easier. Given that such data have been published by others before (e.g. Jang *et al.* 2017, PMID: 29093062, Fig.1D), we perceived it as more ‘novel’ to focus on the inflamed lung for our study. However, we checked in one experiment also the T cell response in the lymph node (**Figure A**), demonstrating that the cytokine response of T cells in the lymph node does not contradict to our lung data (Fig.4B). These lymph node data (**Figure A**) are for the reviewer’s perusal, as we do not propose to include these preliminary data.

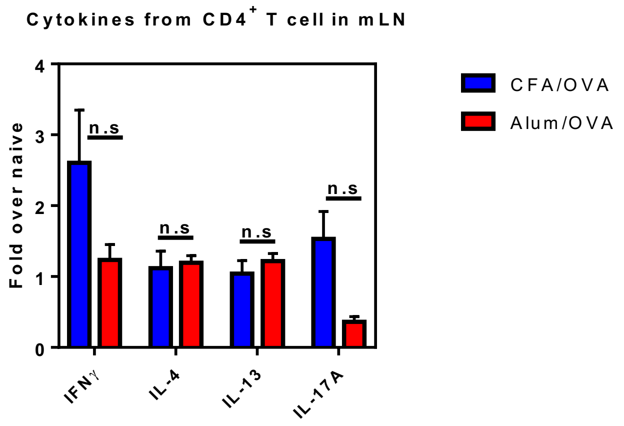

Figure A. Cytokine production by conventional lymph node CD4$^+$ T cells: C57BL/6 mice (naïve group n = 3 mice/group, experimental asthma groups n = 4-5 mice/group) were either immunized three times weekly intraperitoneal (i.p.) with Alum (1 mg/mouse) for eosinophilic asthma or once subcutaneous (s.c.) with CFA (0.5 mg/mL) for neutrophilic asthma along with the model antigen OVA (20 µg/mouse). Three weeks after the first administration, the experimental groups were challenged with OVA (50 µg/mouse) daily for two days. Samples were collected 16-18 hours post-challenge. Cells from mediastinal lymph nodes were stained for CD4$^+$ T cells (Live CD45R/B220$^-$ CD1d/PBS57-tetramer$^-$ TCRβ$^+$ CD4$^+$ cells) to measure the production of indicated cytokine via intracellular cytokine staining. n.s. = not significant. Preliminary data from one experiment is shown.

2b. Fig1E, the cytokine was measured in BALF samples which might add more variability in data. How was the data normalized?

Response: Experimental samples were loaded in duplicates and were analyzed in parallel and together with the samples for the standard curve on the same ELISA plate for each cytokine.

3a. Author should also include the chemokines which are majorly secreted during inflammation either eosinophilic or neutrophilic.

Response: The main aim of our study here is to compare the myeloid compartment during adjuvant-induced eosinophilic and neutrophilic airway inflammation in unprecedented detail side-by-side. We measured IL-5, IL-13, and IFNγ in the BALF to support our conclusion that we can successfully model these two types of airway inflammation. We believe that adding data on the chemokine concentrations in BALF would only marginally strengthen our conclusion. A detailed comparison of multiple chemokines in the BALF of mice with the adjuvant-induced asthma models we employed here has been published before (Bogaert *et al.* 2011, PMID: 21335522, Fig.4 and Dejager *et al.* 2015, #25760421). Furthermore, the expression levels of several chemokines, like CCL2/MCP1, CCL3/MIP1α, CCL5/RANTES, or CCL11/eotaxin, peak early (4h) after the allergen challenge and are decreased by 24h (Lloyd *et al.* 2002, #11872089), the time-point for our analysis. In contrast cytokines, like IL-5, peak 24h after the challenge (Lloyd *et al.* 2002). These data suggest that the 24h time-point of our protocol is suboptimal for the detection of chemokines in the BALF. In light of these points, we therefore feel that adding BALF

chemokine data does not justify the significant delay in publication that would be the result of repeating these experiments twice more.

3b. ELISA data showing IgG2b or IgG2b could be removed from figure 1.

Response: As suggested by the reviewer, we moved the graphs for IgG2b and IgG2c from figure 1F to the new supplemental figures S1C.

4. Figure4B, author differentiated the Th1/Th2/Th17 cell and NKT1/NKT2/NKT3 cell using combination of cell specific surface markers and transcription factors. At same time author have measured the cytokine in the lung samples. How will author explain about the source of cytokines as Th cells as well as NKT cells express T cell markers like CD3 or CD4?

Response: The reviewer is correct that just using CD4 and the transcription factors would not allow us to distinguish conventional $CD4^+$ T cells from the *i*NKT cells. However, the flow cytometric analysis of the intracellular cytokines used for figure 4B also included fluorescently-labelled CD1d/PBS57-tetramers, which specifically mark *i*NKT cells. CD1d/PBS57-$tetramer^+$ cells were excluded from the analysis of the conventional $CD4^+$ T helper subsets. Therefore, we are confident that the data in figure 4B only reflect the cytokine production of conventional $CD4^+$ T cells. However, we failed to include this important information and apologies for this omission. This explanation was added now to the figure legend of figure 4 in the revised version of the manuscript.

5. During lung cell preparation complete RPMI media was used. What is meaning of "complete RPMI media"?

Response: The 'complete' refers to the RPMI medium with all the supplements. The explanation of the supplements was inadvertently lost during the preparation of the manuscript. We thank the reviewer for pointing this mistake out to us. We added now the missing explanation to the revised text.

6. Figure1D and Figure3D with poor quality.

Response: We thank the reviewer for pointing this quality issue out to us. We replaced the figures with files of higher resolution for the revised version of the manuscript and hope that this improves the readability of the figures.

We believe that we have fully addressed all the reviewers' concerns and hope that our revised manuscript is now suitable for publication in *PLOS ONE*.

Please do not hesitate to contact me if you had any questions.

Yours sincerely,

G. Wingender

Gerhard Wingender
